# A Model of Trust Processes in Borderline Personality Disorder: A Systematic Review

**DOI:** 10.1007/s11920-023-01468-y

**Published:** 2023-10-27

**Authors:** Emanuele Preti, Juliette Richetin, Anita Poggi, Eric Fertuck

**Affiliations:** 1 Department of Psychology, University of Milano-Bicocca, Piazza dell’Ateneo Nuovo, 1, 20126 Milan, Italy; 2 City College of the City University of New York, New York, USA

**Keywords:** Borderline personality disorder, Trust, Interpersonal problems, Systematic review

## Abstract

**Purpose of Review:**

Unstable relationships are a core feature of borderline personality disorder (BPD). Impairments in trust processes (i.e., appraisal and learning regarding others’ trustworthiness) can subserve interpersonal problems associated with BPD, but the determinants, mechanisms, consequences, and variations in trust impairments among individuals with BPD remain poorly characterized. Thus, a better understanding of such impairments could help target interventions that address the interpersonal problems of individuals with BPD beyond emotion dysregulation, impulsivity, and aggression.

**Recent Findings:**

We conducted a pre-registered systematic review of empirical studies on trust processes and BPD features (*k* = 29). Results are organized around a heuristic model of trust processes in BPD comprising the following stages: developmental factors, prior beliefs and dispositions, situation perception, emotional states, trust appraisal, behavioral manifestations, and trust learning.

**Summary:**

Based on the synthesis of the findings, we recommended directions for future research and clinical assessment and intervention, such as managing trust during the early stages of therapy and considering improvements in trust processes as a central mechanism of change in treating individuals with BPD.

**Supplementary Information:**

The online version contains supplementary material available at 10.1007/s11920-023-01468-y.

## Introduction

The ability to appraise who is trustworthy and update one’s appraisal of others’ trustworthiness accurately, flexibly, and efficiently in the face of new information about them is essential in navigating the spectrum of human relationships. Individuals with borderline personality disorder (BPD) exhibit extreme distress and confusion in social environments and display behaviors that indicate impairments in appraising others’ trustworthiness. BPD is a multifaceted clinical condition characterized by marked impulsivity and a pattern of instability that influences self-image, interpersonal relationships, and affects [1]. Although disturbed interpersonal relationships historically have been recognized as a core dimension of BPD [2, 3], empirical research has been tilted toward investigating impulsivity and affective instability. In the past 10 years, however, empirical research has increasingly focused on interpersonal difficulties in BPD [4–6].

From the perspective of psychopathology research, dysfunctions in “social processes” [7] underlie a range of symptoms and traits of BPD and can be considered core deficits that interact with emotion dysregulation (for reviews, see [7–9]). There are two primary rationales for dissecting trust processes in individuals with BPD. First, interpersonal dysfunctions in BPD often are precipitants to high-risk behaviors (i.e., suicide attempts and non-suicidal self-injury; [10]). Second, many BPD symptoms (e.g., self- and other-aggressive behaviors, emotional instability) primarily occur in turbulent relationships [11].

Furthermore, trust impairments in BPD may manifest in adverse countertransference reactions [4], difficulties in developing and maintaining solid therapeutic alliances [12], and “splitting” clinicians into idealized or persecutory camps [13]. Different theoretical and clinical approaches have conceptualized the interpersonal domain as a particular focus of psychotherapeutic intervention [14–16]. Premature treatment termination may result from impaired trust appraisal of clinicians. A personality disorder (PD) diagnosis (of which BPD is the most frequent) predicts early drop-out of psychotherapy [17].

Developmental psychology [18] and social [19], cognitive, and affective neurosciences [20] have recently made strides in unraveling the psychological and neural mechanisms subserving interpersonal trust processes. However, a conceptual framework that integrates trust appraisal, decision-making, and learning into a comprehensive model of trust processes remains incomplete. We argue that trust processing is a multi-stage and iterative process, and that those with BPD may exhibit a unique constellation of impairments at different stages of these processes [13].

### A Model of Trust Processes and Borderline Personality Disorder

Diverse conceptualizations of trust processes prevail in social psychology, sociology, anthropology, economics, and political sciences [21–23]. Across these frameworks, *trustors* are agents who depend on others’ behaviors for their goals and objectives. *Trustees* are the agents who can react in a trustworthy or untrustworthy way to trustors’ goals and actions. Trust is relevant when trustors depend on trustees’ action(s) to achieve their goals and objectives [23, 24]. When acting on their trust, trustors make themselves dependent upon trustees’ actions to obtain their goals [23].

The integration of elements from these different frameworks informed our formulation of a conceptual model. In accordance with personality psychology, we assume that the ability to develop and maintain trust is an individual difference that may vary within persons and depends on the context and the nature of the relationship between a particular trustee and trustor dyad. In accordance with the social psychological framework, we emphasize the social environment’s effect on individuals’ trust behaviors. We also presume that trust evolves in interpersonal relationships both developmentally in light of the quality of early attachment experiences and within particular relationships later in life. We posit that trustors can learn to trust based on social reward or punishment patterns in interactions with trustees. From economic psychology, we acknowledge individuals may have an intrinsic self-interested motivation during interpersonal exchanges. Future investigations about trust’s psychological underpinnings in mental health disorders such as BPD may benefit from such an integrative model, including contributions from diverse fields.

**Fig. 1:**
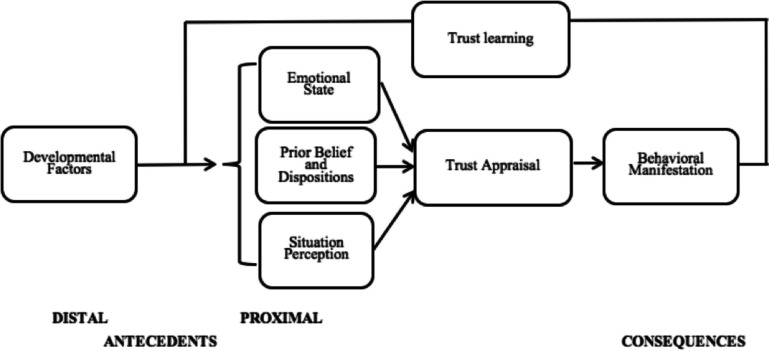
A multi-stage, integrative model of trust processes

Deciding whether to trust others, take action following such a decision, and then re-evaluate the degree of trust in a relationship implies an iterative, multi-step process. We thus propose a heuristic for a multi-stage model of trust processes, assuming that the different stages of the model interact with each other in determining the unfolding of trust processes. We will utilize this model to characterize normative and atypical BPD trust processes at each normative stage (see Fig. 1).

We organized our review according to the different stages of the model. First, we synthesize the literature on the first stage: *distal and proximal antecedents of trust attribution* (i.e., factors that help build and support the appraisal of others’ trustworthiness). Considering *distal antecedents*, in typical development, children experience trusting, attuned behavior from their caregivers, implying that the trustee (i.e., the trusted person, caregiver) generally does not violate trust expectations. Attachment theory posits that such favorable experiences lay the foundation for positive internal working models of the infant’s relationship with others that later lead to an accurate appraisal of trustworthiness in others [25]. According to Erikson [26], trust emerges from responsive and consistent caregiving experiences. Conversely, when infants are mistreated, abused, or must wait extensively for the attachment figure to comfort them, they can develop legitimate mistrust. As a consequence of positive caregiving experiences, infants with secure attachments differ from infants with insecure attachments (i.e., avoidant or anxious-ambivalent) because they expect to trust attachment figures during stressful times [27]. Such attachment styles extend into adolescence and adult years [28], driving securely attached infants to develop trusting relationships in adulthood.

Our model then considers three categories of proximal antecedents. First, regarding *prior beliefs and dispositions*, early findings on typical individual tendencies to trust others are inconsistent. According to Botsford et al. [29], individuals typically assume others to be potentially reliable and trustworthy, leading to mutually beneficial interpersonal exchanges. Nonetheless, approximately 70% of participants in the European and World Values Surveys did not agree with the statement “most people can be trusted” (European Values Study Group and World Values Survey Association, 1999–2002). Although general trust in others is a positive predictor of self-rated health [30–33], distrustful beliefs can be functionally protective according to the adaptative-evolutionary approach. Suspecting a trustee increases one’s vigilance in vulnerable situations [22, 34]. Despite such mixed findings on trust dispositions in the general population, the development, maintenance, and activation of dysfunctional prior beliefs about others’ untrustworthiness comprise one plausible proximal cause of trust impairments in the BPD population.

The second proximal antecedent considered in our model is *situation perception*. Social behavior cannot be dissociated from the situation [35]. Personality traits can partially determine behavior, but what people do also depends critically on their circumstances [36]. The perception of a trust-relevant social situation might modulate trust behavior. Thus, trust impairment may result from the interaction between dysfunctional personality characteristics, dysfunctional beliefs, dispositions, and modulating social contexts. Regarding BPD, several studies investigated whether trust impairments could relate to dysfunctions in appraising trust-relevant interpersonal situations.

The last antecedent of trust appraisal in our model is related to the trustor’s *emotional state*. Besides early emotional experiences, a trustor’s emotional state in the here and now also may interfere with trustees’ trust-related interactions. For example, a trustee’s mood and emotional state contribute to the ongoing experience of trust [37]. Psychodynamic models regarding the development of dysfunctional interpersonal behaviors in BPD, such as mentalization theory [38] and object relations theory [15], and cognitive-behavioral theories (e.g., Biosocial Model; [39]), converge on the critical role of positive and negative emotions on trust appraisal.

We next analyze contributions in the model’s second stage: *Trust appraisal*. Appraising others’ trustworthiness is such a relevant judgment for interpersonal exchanges that people, on average, make initial trust appraisals of others based on visual facial morphology after only 100 ms [40].

Our model also considers how trust is expressed through decision making regarding engaging in behaviors towards trustees. Researchers often have used game theory procedures to investigate the behavioral manifestations of trust in controlled experimental conditions (i.e., *decision-making in social contexts*). Game theory considers the roles of emotions, mistakes, limited foresight, doubts about how intelligent others are, etc., in the study of decision-making around trustees’ trustworthiness [41].

Finally, we examined the iterative process of updating trustworthiness appraisals and social behaviors according to novel trust-relevant interactions and stimuli, i.e., *the trust learning process.* Trust learning refers to “learning whom to trust and when to revise trust attributions” [42•]. A successful trust learning process results in adaptive updates in the appraisal of others’ trustworthiness due to exposure to trust-relevant interpersonal interactions. Conversely, rigidity and inflexibility in trustworthiness appraisal may signal a failure in trust learning processes. For instance, in economic game procedures, accurate trust learning corresponds to an increase in the likelihood of positive and cooperative interactions in future transactions after the trustors’ investment is reciprocated with cooperation. When trustees violate trustors’ trust by defecting, the probability of future trust decreases significantly [37].

## Method

**Fig. 2:**
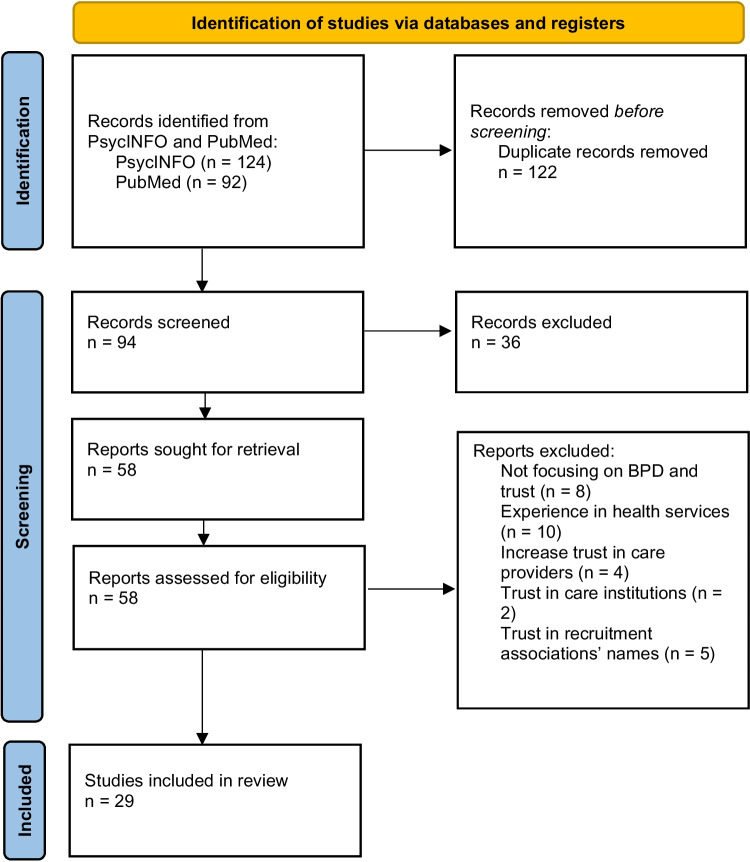
Flow diagram of study selection

We registered our review protocol with PROSPERO (CRD42019125457), systematically reviewed the literature related to trust and BPD, and reported our methods and results while following PRISMA recommendations. We used the electronic databases PsycINFO and PubMed to locate studies that address the topic, searching for specific keywords in the title or abstract [“trust” OR “trustworthiness” AND “borderline personality disorder”]. We conducted the literature search of databases in July 2023^1^ and identified 124 records in PsycINFO and 92 in PubMed (for the selection flow diagram, see Fig. 2). A full description of the search procedure is in Supplement 1. We ended up with a final set of 29 research reports.

The key characteristics of all 29 studies included are reported in Table 1 (Table S1 contains all the details of the 29 studies included).

**Table 1:** Empirical studies addressing trust impairment in borderline personality disorder

	**Study**	**Sample**	**Key findings**
Developmental factors	Ebert et al. (2013) [44]	13 BPD 13 Controls	Only in BPD participants in the oxytocin condition childhood trauma correlated with trust behaviors
	Orme et al. (2019) [43•]	322 adolescent inpatients	Negative association between BPD and trust in mothers and fathers
Prior beliefs and dispositions	Butler et al. (2002) [49]	84 BPD 102 Other PDs	“I cannot trust other people” is the most discriminative belief for BPD
	Botsford et al. (2019) [29]	41 BPD 30 MDD 31 SAD 236 Controls	BPD patients display lowest levels of interpersonal trust
	Graves et al. (2021) [50]	87 BPD 197 Psychiatric patients 165 Controls	BPD patients displayed lowest levels of emotional trust
	Miano et al. (2013) [52]	95 Non-clinical	The high-BPD group’s higher untrustworthiness was mediated by RS
	Richetin et al. (2018) [54]	125 Non-clinical	Only emotional RS mediate the effect of BPD on trust appraisal
	Bartz et al. (2011) [47]	14 BPD 13 Controls	Oxytocin produced more decrease in trust and cooperation in BPD. Effects driven by anxious attachment and RS
Situation perception	Miano et al. (2017) [55••]	31 BPD 36 Controls	BPD reported less trust toward partners only after threatening discussions
	Preuss et al. (2016) [56]	17 BPD 36 Controls 24 MDD	BPD had more inconsistent behavior only in the social conditions: social trust game and punishment game
Emotional state	Hula et al. (2017) [58]	55 BPD 38 Controls	BPD participants were less aware of guilt and irritation than controls
	Roberts et al. (2018) [59]	284 Non-clinical	Acetaminophen reduced behavioral mistrust at high levels of BPD
	Masland and Hooley (2019) [62]	77 Non-clinical	Trust appraisal influenced by negative priming in BPD more than controls
Trust appraisal	Fertuck et al. (2013) [63]	17 BPD 19 Controls	BPD show an increased response bias in trustworthiness appraisal
	Nicol et al. (2013) [64]	20 BPD 21 Controls	BPD judged faces as less trustworthy
	Houben et al. (2018) [66••]	30 BPD 28 Controls	Lower appraisal of others’ trustworthiness in daily life in BPD
	Fertuck et al. (2019) [65•]	16 BPD 17 Controls	Lower trustworthiness appraisal in BPD. Lower activity in prefrontal cortex related to bias intensity
	Biermann et al. (2022) [67]	75 BPD 67 Controls	Untrustworthiness bias in BPD, regardless of presence of face mask
Behavioral manifestations	King-Casas et al. (2008) [57••]	55 BPD 38 Controls	BPD more likely to cause cooperation ruptures and lower coaxing behaviors
	Unoka et al. (2009) [68]	25 BPD 25 Controls 25 MDD	BPD participants transferred less money during the trust game. No differences in the lottery game
	Saunders et al. (2016) [70]	20 BPD 20 Controls	BPD participants failed in building cooperative relationships
	Hepp et al. (2018) [73]	26 BPD 26 Controls	No differences between BPD and controls in money shared
	Lévay et al. (2021) [74]	30 BPD 30 Controls	No differences in money shared. BPD reported more selfish other’s expectations
	Niedtfeld and Kroneisen (2020) [69]	51 BPD 50 Controls	BPD participants transferred less money during the trust game only when playing with trustworthy faces
Trust learning	Franzen et al. (2011) [75]	30 BPD 30 Controls	No differences in investment behaviors according to others’ facial expressions
	Bo et al. (2017) [78]	25 BPD adolescents	Pre-post changes (after MBT group treatment) in trust in parents and peers
	Fineberg et al. (2018) [42•]	20 BPD 23 Controls	Lower trust learning scores in BPD, even if they weighted more social cues
	Abramov et al. (2020) [76]	234 Non-clinical	BPD associated with greater decline in investments during trust-formation
	Abramov et al. (2022) [77]	234 Non-clinical	BPD investments partly explained by feelings rejected and self-protective beliefs

*BPD* borderline personality disorder, *PD* personality disorder, *MDD* major depressive disorder, *SAD* seasonal affective disorder

## Results

### Distal Antecedents: Developmental Factors

This section outlines studies suggesting developmental risk factors that may facilitate impairments in trust processes in adult individuals with BPD. Orme et al. [43•] tested whether a relative lack of *epistemic trust* in childhood was associated with BPD symptoms in a sample of adolescent BPD inpatients admitted to a psychiatric unit. They found a significant negative correlation between BPD symptoms at admission and self-reported trusting state toward participants’ mothers and fathers.

Ebert et al. [44] hypothesized that childhood trauma is a risk factor for developing dysfunctional behavioral manifestations during a trust game (TG) procedure in individuals with BPD. Additionally, they were interested in the role of oxytocin. While oxytocin administration is usually associated with higher interpersonal trust [45, 46], the administration of this neuropeptide seems to have a paradoxical effect in BPD (e.g., see below [47]). Increased oxytocin seems to reduce trusting behaviors in individuals with BPD (but not controls), and such a decrease is more important for individuals with BPD who reported greater early parental emotional neglect. Overall, results suggest that developmental factors, such as emotional neglect experiences and lack of trust in parents (as a proxy of epistemic trust), are distal risk factors for trust issues in adults with BPD.

### Proximal Antecedents

#### Prior Beliefs and Dispositions

According to investigations on core dysfunctional beliefs in personality disorders [48], mistrust represents a specific feature of BPD. Butler et al. [49] used the Personality Belief Questionnaire (PBQ) [48] and found that the item that best distinguished BPD from other personality disorders was, “I cannot trust other people.” Such a belief represents a dysfunctional global expectation of others’ trustworthiness. BPD patients self-reported lower interpersonal trust levels than non-clinical controls and patients with MDD or seasonal affective disorder [29]. Similarly, comparing adolescents with BPD with adolescents with other psychiatric conditions and a non-clinical sample, the BPD group self-reported the lowest level of emotional trust [50].

Other studies investigated the role of other dispositions, such as rejection sensitivity (RS, [51]), whose high levels may influence the positive association between BPD and impaired trust processes. Individuals with strong BPD features might be less inclined to trust others because of their concerns and anxiety about the possibility of being rejected or abandoned. In a sample of undergraduates, RS fully mediated the negative association between BPD features and facial appraisal of untrustworthiness [52]. More precisely, only emotional RS components (anger and anxiety about being potentially rejected), not the cognitive one (expectations) (see [53]), mediated the association between BPD features and facial appraisal of others’ trustworthiness [54].

Some studies show that oxytocin administration results in increased trusting behavior during economic games [45]. Bartz et al. [47] hypothesized that individuals with BPD might show an altered response to intranasal oxytocin because its effects on trust and prosocial behavior vary as a function of the relationship representations one possesses. Oxytocin administration in individuals with BPD resulted in expectations of lower cooperation. Conversely, healthy controls showed higher trusting expectations following oxytocin doses than placebo controls. Anxiously attached and rejection-sensitive participants predominantly accounted for these divergent results. Whereas oxytocin generally fosters trust exchanges, the effect is reversed when oxytocin interacts with attachment insecurities and personality traits of rejection sensitivity that are common in BPD patients.

In summary, studies support the model’s stage about the role of dysfunctional prior beliefs regarding others’ trustworthiness for trust impairments in BPD, indicating greater mistrustful beliefs in BPD trustors and pointing to the role of prior dispositions in the impairments in trust processes in BPD.

#### Perception of the Situation

Two studies using different methodologies demonstrate that BPD patients show state-dependent trust impairments connected to specific situations.

On the one hand, women with BPD did not differ from healthy controls on their partners’ perceived trustworthiness after a neutral conversation, but their trust in their partners decreased after personal or relationship-threatening discussions [55••]. On the other hand, Preuss et al. [56] tested whether social, compared with non-social, situations could activate untrustworthiness appraisal bias more easily in individuals with BPD, using different tasks. BPD participants demonstrated significantly less-consistent behavior (i.e., more investment variability) than the healthy control and MDD group in the social conditions (trust game and punishment game). However, the BPD group did not exhibit such volatility in the non-social conditions.

#### The Impact of the Trustors’ Emotional State on Trust Processes

King-Casas et al. [57••] used a functional magnetic resonance imaging approach to examine differences between BPD and control groups both behaviorally and neurally during a trust game. Hula et al. [58] analyzed the King-Casas’ data set, adopting an alternative computational model that allows for inferences about three experiences relevant to trust: risk aversion, irritation, and guilt. The authors showed that BPD trustors experienced less guilt and more irritation than healthy control trustors during the economic game. The authors labeled trustees with low guilt-proneness and high irritation as “perilous individuals” who deliberately exploit the trustee and create problematic interactions. Perilousness was more common in the BPD sample compared with controls. Furthermore, perilous individuals were more likely to interpret cooperative situations negatively and were less prone to establish cooperative interchanges or repair cooperation ruptures. Moreover, like perilous individuals, individuals with BPD showed increased irritation from unpleasant interactions during economic exchanges. These results highlight the effects of the trustor’s emotional state in determining mistrust in BPD.

Roberts et al. [59] reported that the administration of acetaminophen, a pain reliever, reduces behavioral mistrust (i.e., low investment) exhibited by participants with high levels of BPD features during a Trust Game procedure. There were similar rates of untrustworthy expectations in individuals with high and low BPD features regardless of acetaminophen or placebo administration. The authors speculated that the decrease in behavioral mistrust in the acetaminophen condition among individuals with high BPD features was due to a reduction in negative emotional affect related to possible unpleasant outcomes in interpersonal interactions (and not due to changes in expectations). Previous evidence showed that acetaminophen reduced negative affective responses (e.g., to rejection; [60, 61]).

Masland and Hooley [62] examined the influence of an emotional prime on trustworthiness appraisal. Non-clinical participants with high versus low borderline features rated unfamiliar faces’ trustworthiness after an affective priming paradigm that exposed them to negative, neutral, or positive images. High-BPD-features individuals showed significantly lower trust appraisal after exposure to negative, neutral, and positive primes than the low-BPD group. However, low- and high-BPD groups showed a significant decrease in trust appraisal after negative emotional primes. Compared with the low-BPD group, negative affective primes influenced appraisal more in the high-BPD group.

To summarize, three studies with different methodologies and samples indicate that individuals with BPD (or higher BPD features) show greater trust impairments with increased state negative affect.

### Trust Appraisal

Considering trust appraisal, the human face is a salient source of interpersonal information. The appraisal of others’ trustworthiness is such a relevant judgment for interpersonal exchanges that people, on average, make initial trust appraisals of others based on visual facial morphology after only 100 ms [40].

Fertuck et al. [63] compared facial trustworthiness and fear appraisal in BPD and healthy controls. Compared with controls, BPD participants rated the trustees’ faces as more untrustworthy, indicating the presence of an untrustworthy response bias to all trust faces. By contrast, no significant differences in sensitivity, discriminability, or bias in fear appraisal emerged. Moreover, BPD participants showed slower RT trustworthiness ratings than controls, especially toward more ambiguously trustworthy faces, while there were no differences in RTs for fear ratings between groups. 

Nicol et al. [64] found similar results comparing participants with BPD vs. controls when assessing whether facial stimuli appraisal of age, distinctiveness, attractiveness, intelligence, approachability, and trustworthiness. BPD participants showed a significantly larger untrustworthiness bias effect than controls in social dimensions appraisal, such as unknown faces’ approachability and trustworthiness. There was no difference in non-social aspects of the appraisal of others between groups. 

Fertuck et al. [65•] replicated the behavioral findings in an fMRI study. Furthermore, BPD participants during untrustworthiness ratings evidenced less activity in the anterior insula and lateral prefrontal cortex than the controls. Such a decrease was proportional to the degree of untrustworthiness bias and impaired discriminability demonstrated by BPD patients and the controls. Individuals with BPD did not show amygdala hyperactivation relative to healthy controls during trustworthiness or fear appraisal. Thus, impaired probabilistic reasoning (linked to prefrontal cortex activity) might be more relevant than hypersensitivity to threatening stimuli (traditionally linked to hyperactivity in the amygdala) in playing a role in trustworthiness appraisal impairments in BPD.

Houben et al. [66••] examined momentary appraisals in a group of individuals with BPD compared to healthy controls in an Ecological Momentary Assessment (EMA) study. Participants were prompted 10 times per day for 8 days to answer questions about their momentary emotions and appraisal of their living situations. Compared to healthy controls, BPD patients experienced lower levels of trustworthiness in trustees in their daily lives.

Finally, Biermann et al. [67] investigated the impact of face masks on trust judgment of faces. Face masks were associated with an overall drop in trust. Irrespective of the presence of a face mask, BPD participants reported lower trust ratings of faces than healthy controls.

In summary, five studies consistently documented a specific bias in lower trust appraisals of trustors’ facial stimuli among BPD patients compared with healthy controls. Furthermore, in our previous section (prior beliefs and dispositions), three additional studies reported results that point in the same direction [52, 54, 62]. This effect is also present when examining BPD patients’ daily lives [66••].

### Behavioral Manifestations: Interpersonal Cooperation and Repair of Ruptures in Cooperation

Several researchers have investigated atypical trust manifestations in the BPD population using game theory procedures. King-Casas et al. [57••] used a trust game procedure recording neural activation in a BPD and control group. The authors focused on the capacity to sustain a mutually rewarding, cooperative social exchange (*vs.* cooperation ruptures) and the ability to repair non-cooperative interactions (“coaxing” behaviors, i.e., when a trustor repays a large part of the investment to the trustee to signal their trustworthiness and gather more substantial investments on subsequent rounds from trustees). Compared with the controls, BPD patients were more likely to initiate cooperation ruptures by sending rejecting social signals. Moreover, BPD trustors had lower rates of coaxing behavior to repair the cooperation ruptures than the controls. Furthermore, anterior insula activity is related to the violation of social norms perception in non-clinical samples, but this was not the case in the BPD sample. Because BPD group showed no insula activation, the authors attributed BPD patients’ low investment behaviors to a lack of sensitivity (assessed via insensitive insula activity) from social norms violations. Furthermore, a lack of insula activation in the BPD group may occur because of dysfunctional beliefs’ top-down influence on neural activity, such as holding negative expectations about social partners.

Unoka et al. [68] replicated King-Casas et al.’s [57••] behavioral effect. The authors used a single-trial TG and a risk game in three trustor groups: BPD, major depression disorder (MDD) individuals, and controls. Additionally, before playing the trust and risk games, participants shared their expectations about the games’ outcomes. In the single trial TG, the trustee can share a fair number of monetary units with the trustor (i.e., the participant) or an unfair amount (violating the investor’s trust). By contrast, in the risk game, the number of monetary units the trustees return to the trustor is determined randomly. The BPD group evidenced lower investment rates in the TG procedure than the MDD and control groups but comparable investment rates to the other groups in the risk game. Moreover, BPD had more skeptical forecasts about the TG outcomes and more accurate estimates about the risk game compared with MDD individuals and controls.

Niedtfeld and Kroneisen [69] used a single-trial TG and tested whether a BPD group show altered memory for cooperative versus non-cooperative interaction partners relative to a control group. Female BPD patients and healthy controls played 40 rounds of single-trial trust games interacting with trustworthy and untrustworthy faces in a source memory paradigm. Half of the rounds resulted in cooperative interactions from trustees, whereas target faces (trustees) behaved uncooperatively for the other half. The BPD group invested lower amounts of money for trustworthy targets than controls. For untrustworthy faces (trustors), on the contrary, no differences emerged between the groups. Moreover, the BPD group had significantly more difficulty recalling cooperative targets than controls. No differences emerged for uncooperative targets.

Saunders et al. [70] found cooperation impairments in BPD patients with an iterated form of the prisoner’s dilemma game [71]. The original prisoners’ dilemma comprises participants who choose to cooperate or defect (i.e., keep all monetary units for themselves) for their sole or joint benefit. The iterated version allows for measuring how individuals acquire and maintain reciprocal altruistic behavioral patterns in multiple exchanges. In the iterated version, the rational strategy is to seek cooperation that maximizes both players’ gains. To get the maximum mutual benefit, the trustor should systematically repeat the trustee’s last choice, undertaking a “tit-for-tat” approach to elicit cooperation from social partners [72]. BPD patients were less able to form reciprocally cooperative relationships with social partners (i.e., they did not assume a tit-for-tat strategy) than the controls [70].

However, Hepp et al. [73] did not find significant differences between a group of individuals with BPD and a group of healthy controls in a dictator game in which participants acted as allocators, thus showing no differences in cooperative behavior in BPD.

Lévay et al. [74] found that BPD patients did not differ in their prosocial disposition from healthy controls in a Social Value Orientation task in which participants must decide how to divide sums of money between themselves and a fictive unknown person. Interestingly, BPD participants were more prone to anticipate a selfish decision in money division from the other.

In summary, four out of six studies support that, compared with controls, BPDs show reduced behavioral cooperative and reparative interactions with trustees.

### Trust Learning

In a study [75], trustees expressed happiness or anger during a TG. Trustee fairness was manipulated in the game to display fair vs. unfair behavior. BPD patients showed better performances than control since they could adapt their investments to the actual fairness of the trustee even in the condition with emotional cues, whereas control participants did not show differences in their endowments between fair and unfair trustees when emotional cues were presented. The authors interpreted these results regarding the superiority of Theory of Mind in BPD patients.

Fineberg et al. [42•] investigated the different weights of social and non-social cues in a learning task for BPD participants compared with controls. The task design included five different subphases that varied in terms of volatility and reliability of cues. The participant’s task was to learn the reward probability of social (i.e., partner’s advice during the game) and non-social (i.e., computer’s advice) cues. Learning rates were modeled based on the number of trials occurring between the start of the phase and the engagement of choices consistent with the ongoing condition (i.e., following the advice during stable and reliable stages and not following the advice during volatile or unreliable phases). The authors, examining the transcripts of participants’ answers to debriefing questions, counted the number of times each participant mentioned the confederate. Compared with controls, the BPD group mentioned the confederate more frequently, suggesting more attention paid to and dependence upon social cues. Looking at learning rates during the task, the BPD group learned more slowly than control subjects during all three phases. Furthermore, the BPD group showed slower learning rates in the volatility conditions than the control group.

Abramov et al. [76] implemented a 15-round trust game manipulating trustees’ investment rates with three separate phases: formation of trust, dissolution of trust, and trust restoration. Individuals with high BPD features showed declining trust toward trustees only during the formation of the trust phase. Surprisingly, following trust violation and during the restoration phase with a trustee, trustors with high BPD features showed higher investment rates than individuals with low BPD features. In a reanalysis of these data [77], the authors found that feelings of rejection and self-protective beliefs partly explained these paradoxical effects. These results point to the role of previous beliefs and dispositions in trust learning.

A novel way of considering trust learning processes in BPD is to assess changes in trust appraisal as a consequence of effective psychotherapy. Only one study addresses this issue. Bo et al. [78] investigated changes in trust toward parents and peers as treatment outcomes of MBT group treatment in adolescents with BPD. Although there was no control group, there was significant pre-post changes in trust in peers and parents. Although we should apply caution because these results do not come from a randomized controlled trial, this is the first partial demonstration that BPD adolescents might learn from psychotherapy to trust more their peers and their parents.

## Conclusions

With this review, we aimed to propose a multi-stage, integrative model of trust processes (Fig. 1) to dissect BPD-specific impairments at each stage. The model outlines distal and proximal antecedents of trust appraisal, influencing cooperative behaviors in interpersonal exchanges and trust learning. We examined the variations from typical processes related to BPD, a clinical condition strongly associated with trust impairments, and found preliminary support for significant atypical processes at each stage of the model in BPD.

Regarding distal antecedents, developmental adversities might be associated with trust impairments in BPD patients. This corresponds with research on the impact of developmental factors in the emergence of BPD (e.g., [79]).

Concerning proximal antecedents, dysfunctional beliefs and dispositions, increased sensitivity to situational cues, and greater negative emotional states in BPD trustors predicted trust impairments in BPD patients. Considering a synergistic relationship between stable (i.e., personality traits, prior beliefs, and dispositions), unstable (or volatile, such as the perception of the situation), state (emotional state of the trustor), and contextual factors, we argue for an interactionist perspective, in terms of the significant role of situations in translating personality factors into behaviors, from social cognitive and emotional perspectives. We suggest that situation-behavior contingencies can intensify baseline pathological traits such as untrustworthiness biases and beliefs.

Regarding trust appraisal, studies showed a consistent bias toward untrustworthiness among BPD patients, whereas considering cooperative behaviors, individuals with BPD tend not to trust trustees, leading to more frequent ruptures in cooperation.

In our heuristic model, we suggest that an efficient process of updating our expectations regarding others’ trust is relevant for nurturing adaptive, stable, trusting relationships over time and deciding whom one should avoid. Considering our iterative model, we argue that the quality of trust learning responds to and influences the emotional state, the perception of the situation, the disposition, appraisals of trust, and individuals’ behavior. The contingency approach to personality disorders argues for the importance of considering distal and proximal mechanisms as triggers of the expression of symptoms [80]. The economic game studies provided initial evidence of the relevance of a match between personal dispositions and triggering situations through which BPD patients can show dysfunctional features. In the general population, if dysfunctional beliefs and dispositions about trustees do not meet confirmation in social situations, these can be updated and adjusted to more positive ones. This flexibility in trust learning does not seem to occur readily in individuals with BPD.

### Implications for Further Research

This multi-stage, integrative heuristic model delineating trust processes may guide future research in identifying the steps in which trust impairments occur across different mental disorders. Referring to a single model to systematize trust impairments across other diagnoses may allow for comparing and contrasting at each stage across diagnoses.

By dissecting the components of the trust impairment process into discrete, sequential steps, we may not have fully valued the mutual influences occurring between each stage. Since real-life interpersonal trust dynamics occur continuously, we recommend that readers consider each step of the model presented as potentially influencing the whole iterative process, although the interactions among the different stages of the model need to be tested empirically.

In light of the variety of unique methodologies reported in this review, a need exists for replication. Differences exist in the instruments used to assess BPD features, the population examined, the comparison groups used, and the size of the samples examined (see Table S1). Moreover, future research could focus on overcoming several limitations. For example, the studies mainly used visual stimuli from unknown others. It will be important to replicate original findings using alternative stimuli such as familiar others in immersive contexts. Moreover, almost all the empirical contributions were conducted in non-naturalistic settings. There is one relevant exception [66••], which demonstrated impairments in the appraisal of others’ trustworthiness in the daily lives of BPD patients. Using controlled tasks and stimuli in laboratory research significantly limited ecological validity and generalizability to real life in this research domain. Implementing ecological momentary assessment (EMA) procedures may be useful in testing whether dysfunctional trust appraisal in BPD is linked to uncooperative behavioral outcomes in real life. EMA procedures could help better understand several dimensions: the nature and quality of situational cues triggering lower appraisal of trust, the volatility of trust appraisal, and the dynamics of trust learning with related cognitions and behaviors.

Trust learning is a crucial stage of trust impairment in BPD that merits continued emphasis, as it carries implications for prevention and intervention development. Whereas learning models state that people update expected outcomes of future interactions according to simple reinforcement-learning mechanisms, some studies [42•, 75] show that BPD trustors’ untrustworthiness bias may interfere with these learning mechanisms.

Two studies investigated the role of oxytocin in trust processes. Oxytocin has usually been characterized as a “prosocial drug,” with studies revealing that OXT strengthens cooperation by stimulating trust (e.g., [45]). However, other results [44, 47] seem to point towards a paradoxical effect of oxytocin in BPD. These results are consistent with other investigations (e.g., [81]) that hypothesize that oxytocin might act as a modulator of social interactions rather than as a “prosocial” drug independent of context. Thus, further research is warranted exploring the role of oxytocin and other neurobiological parameters in trust processes in BPD.

Finally, evaluating the degree to which these impairments are specific to BPD or could apply to other PDs and psychiatric disorders is essential. Few empirical studies have investigated the link between trust impairment and PDs other than BPD, yet trust impairments might also be relevant in other disorders (e.g., antisocial personality disorder, paranoid personality disorder, or schizotypal personality disorder; see [82]).

### Clinical Implications

To our knowledge, no study has empirically investigated the differences in trust processes across different forms of psychopathology. The framework in this systematic review may facilitate investigations of trust processes across various clinical disorders. Such knowledge might inform a more accurate evaluation and clinical management of trust impairments across conditions by suggesting how to tailor treatments to specific individual impairments in trust processes.

Regarding clinical interventions more broadly, future investigations could evaluate whether trust impairments predict individuals with BPD’s non-completion of treatment. Trust impairments likely influence BPD patients’ medication non-compliance and inconsistent therapy engagement [83]. Clinicians and individuals with BPD patients view trust as a crucial therapeutic relationship element predictive of positive clinical outcomes [84].

More precisely, five major treatments have been established as evidence-based treatments (EBTs) for BPD: dialectical behavior therapy (DBT), mentalization-based therapy (MBT), transference-focused psychotherapy (TFP), schema-focused therapy (SFT), and systems training for emotional predictability and problem-solving (STEPPS) [85]. To varying degrees, all prominent, evidence-based treatments for BPD incorporate strategies and techniques to manage and address trust impairments in BPD. According to a recent contribution, these EBTs for BPD share several common factors: a *coherent* model of treatment, *consistent* interventions rooted in the model of treatment, *continuity* in the treatment model among its practitioners, and quality *communication* between the patient and treator(s) — also known as the four “C”s [86]. While EBTs for BPD mature, the proposed framework model might guide future investigations toward a better understanding of best practices to engender patients’ accurate and flexible appraisal of their clinician’s trustworthiness at all stages of treatment.

In conclusion, using a multi-stage, integrative heuristic model of trust processes as a framework for dissecting how BPD patients express trust impairments, we found evidence of atypical phenomena in the BPD population (or individuals with high BPD features, in the case of non-clinical studies) for each stage. We found robust literature with empirical evidence that BPD patients: (1) tend to report adversities in trusting their caregivers during the earliest developmental stages; (2) tend to rely on a rigid set of dysfunctional beliefs about trustees' untrustworthiness; (3) tend to develop negative trustworthiness bias that applies to trustees with rigidity; (4) tend to exhibit high sensitivity to signs of trust ruptures in with trustees; (5) tend to have intense negative emotions when they perceive a trust rupture with a trustee; (6) tend not to effectively repair ruptures in cooperation with trustees; (7) tend to exhibit impaired flexibility and updating regarding trustee’s trustworthiness based on new, real-time, trust relevant information with trustors. As the reviewed studies take into account separate stages of the model, the interactive nature of the model need to be further investigated.

The current state-of-the-art empirical confirmations of these different stages still need to be more thoroughly elaborated and investigated as the research base is relatively limited. We provided an initial integrative model to organize the theoretical, methodological, and clinical frameworks that empirically studied trust processes in BPD that may elicit subsequent research in this area.

### Supplementary Information

Below is the link to the electronic supplementary material.

Supplementary file1 (DOCX 35 KB)

Supplementary file2 (DOCX 53 KB)

## Footnotes

1 Our initial registered PROSPERO protocol limited the search to July 2020. However, we decided to update our search to the most recent date.
