# Supplementary material for: A Model of Trust Processes in Borderline Personality Disorder: A Systematic Review
Source: Curr Psychiatry Rep. 2023 Oct 27;25(11):555–67. doi: 10.1007/s11920-023-01468-y (PMC10654201; doi:10.1007/s11920-023-01468-y)
Supplement: Supplementary file 1 — Supplementary file1 (DOCX 35 KB) [file 11920_2023_1468_MOESM1_ESM.docx]

Supplement 1. Detailed description of the methods

We conducted a systematic review of the literature related to trust and BPD, registered our review protocol with PROSPERO (CRD42019125457), and report our methods and results, while following PRISMA recommendations. We used the electronic databases PsycINFO and PubMed to locate studies that address the topic, searching for specific keywords in the title or abstract [“trust” OR “trustworthiness” AND “borderline personality disorder”]. We conducted the literature search of databases in July 2023^[[1]](#footnote-2)^ and identified 124 records in PsycINFO and 92 in PubMed (for the selection flow diagram, see Figure 2). We removed duplicates (*k* = 122) and screened the remaining papers’ abstracts (*k* = 94).

Our inclusion criteria were: (1) studies using self-report questionnaires or structured clinical interviews for BPD diagnoses or traits (e.g., a structured clinical interview for DSM-IV axis II; (First et al., 1995); Borderline Personality Disorder Checklist; (Giesen-Bloo et al., 2006); Personality Assessment Inventory; (Morey, 2004); and (2) studies published or accepted for publication in peer-reviewed journals. Our initial exclusion criteria were: (1) studies coming from fields other than psychology (e.g., economics), if these studies did not include individuals validly diagnosed with BPD or measures of BPD traits; (2) studies that did not apply valid and reliable assessment of BPD or BPD traits (i.e., self-report questionnaires or clinical interviews); (3) studies focusing on organizational (and not interpersonal) trust; (4) studies reported in a language other than English; and (5) studies that did not report original quantitative empirical data allowing to quantify trust processes. During this step, we excluded reviews (*k* = 8), qualitative studies (*k* = 8), dissertation abstracts (*k* = 5), clinical illustrations (*k* = 5), commentaries (*k* = 4), pilot studies (*k* = 2), and non-English articles (*k* = 4).

We screened the remaining 58 articles’ content and excluded studies that addressed phenomena linked to trust issues unrelated to our scope. More precisely, we excluded: a) studies not focusing on BPD and trust (*k =* 8); b) studies reporting on users, families, or care providers’ experience in health services and their trust in them or towards BPD individuals (*k* = 10); c) studies focusing on how to increase trust in care providers other than psychotherapists, such as nurses or social assistants (*k* = 4); and d) studies focusing on trust in care institutions (*k* = 2). We also excluded papers that mentioned “trust” within the names of associations for the recruitment of specialists or participants to study (e.g., National Health Service Trust) (*k* = 5). We ended up with a final set of 29 research reports.

In Table S1, for all 29 studies included, we reported the stage of the model to which the study refers, the sample size, the assessment of BPD features, the task that participants performed, and the key findings.

1. Our initial registered PROSPERO protocol limited the search to July 2020. However, we decided to update our search to the most recent date. [↑](#footnote-ref-2)
