# Supplementary material for: A Model of Trust Processes in Borderline Personality Disorder: A Systematic Review
Source: Curr Psychiatry Rep. 2023 Oct 27;25(11):555–67. doi: 10.1007/s11920-023-01468-y (PMC10654201; doi:10.1007/s11920-023-01468-y)
Supplement: Supplementary file 2 — Supplementary file2 (DOCX 53 KB) [file 11920_2023_1468_MOESM2_ESM.docx]

**Table S1.** Empirical Studies Addressing Trust Impairment in Borderline Personality Disorder

| **Study** | **Stage** | **Sample** | **BPD Assessment** | **Task** | **Key Findings** |  |
| --- | --- | --- | --- | --- | --- | --- |
| Ebert et al. (2013) | Distal antecedents:  Developmental factors | *N* = 26  BPD *n* = 13  Control *n* = 13  Random assignment to placebo or oxytocin condition. | Structured  clinical interview for DSM-IV (SCID; German  version by Wittchen et al., 1997) | Trust game  assessment of childhood trauma | Only in BPD participants and only in the oxytocin condition did childhood trauma scores correlate with trust behaviors |  |
|  |  |  |  |  |  |  |
| Orme et al. (2019) | Distal antecedents:  Developmental factors | *N* = 322 inpatient adolescents | Borderline personality features scale for children, child report (BPFS-C; Crick et al., 2005)  Borderline personality features scale for children, parent report (BPSF-P; Chang et al., 2011)  Child interview for DSM-IV borderline personality disorder  (CIBPD; Zanarini, 2003) | Inventory of Parent and Peer Attachment (IPPA) | A negative association was found between BPD measures and adolescent trust in mothers and fathers |  |
| Butler et al. (2002) | Proximal antecedents:  prior beliefs and dispositions | *N* = 288  BPD *n* = 84  Other personality disorder *n* = 102 | Structured clinical  interview for the DSM-III-R (SCID-II; Spitzer et al., 1990) | Personality Belief Questionnaire | “I cannot trust other people” is the most discriminative belief, significantly higher in BPD compared with OPD participants. |  |
| Botsford et al. (2019) | Proximal antecedents:  prior beliefs and dispositions | *N* = 338  BPD *n* = 41  Major depression disorder *n* = 30  Seasonal affective disorder *n* = 31  Healthy controls *n* = 236 | SCID-II | Interpersonal Trust Scenario Questionnaire | BPD patients display lower levels of interpersonal trust compared with non-clinical controls and also patients with MDD. |  |
| Graves et al. (2021) | Proximal antecedents:  prior beliefs and dispositions | *N* = 445 adolescents  BPD *n* = 87  Psychiatric controls *n* = 197  Healthy controls *n* = 165 | CIBPD (Zanarini, 2003)  (BPFS-C; Crick et al., 2005) | Children’s Generalized Trust Beliefs Questionnaire | BPD patients displayed lower levels of emotional trust compared with non clinical controls and also psychiatric controls. |  |
| Miano et al. (2013) | Proximal antecedents:  prior beliefs and dispositions | *N* = 95  Non-clinical population | SCID II Screener for Personality Disorders, Version 2.0 (First et al., 1997). | Facial appraisal on 17 trait dimensions (including untrustworthiness and trustworthiness) | The High-BPD group shows higher untrustworthiness bias than Low-BPD.  RS as a mediator of the effect of BPD features on trust appraisal. |  |
| Richetin et al. (2018) | Proximal antecedents:  prior beliefs and dispositions | *N* = 125  Non-clinical population | Borderline Personality Disorder Checklist (BPDCL;  Giesen-Bloo et al., 2006) | Facial trust appraisal | Only emotional components of RS (anger and anxiety) mediate the effect from BPD features on trust appraisal  No mediation of cognitive component (expectation) |  |
| Bartz et al. (2011) | Proximal antecedents:  prior beliefs and dispositions | *N* = 27  BPD *n* = 14  Healthy controls *n* = 13  Random assignment to placebo or oxytocin condition. | SCID-II | Economic game | oxytocin produced more divergent effects in BPD participants than controls: decrease in trust and cooperation.  Divergent effects were driven by anxious attachment and rejection sensitivity (peculiar to BPD) |  |
| Miano et al. (2017) | Proximal antecedents:  situation perception | *N* = 67 couples  BPD *n* = 31 (women diagnosed with BPD)  Controls *n* = 36 | SCID-II | Evaluation of partners’ trustworthiness after discussion of neutral, personal, or relationship-threatening topics | BPD couples were significantly different from controls only after threatening discussions. |  |
| Preuss et al. (2016) | Proximal antecedents:  situation perception | *N* = 77  BPD *n* = 17  Healthy controls *n* = 36  Major depression controls *n* = 24 | Zanarini Rating Scale for Borderline Personality  Disorders (ZAN-BPD; Zanarini, 2003) | Social trust game  Non-social trust game  Punishment game  Dictator game  Cooperation game | BPD participants had more inconsistent behavior than controls only in the social conditions social trust game and punishment game |  |
| Hula et al. (2017) | Proximal antecedents:  emotional state | *N* = 93  BPD *n* = 55  Controls *n* = 38 | DIPD-IV | Multiple-round trust game | BPD participants were less aware of guilt and irritation than controls. |  |
| Roberts et al. (2018) | Proximal antecedents:  emotional state | *N* = 284  Non-clinical population | Personality Assessment Inventory Borderline Features Scale (PAI-BOR; Morey, 2004). | Economic trust game | Acetaminophen reduces behavioral mistrust at high levels of BPD features. |  |
| Masland & Hooley (2019) | Proximal antecedents:  emotional state | *N* = 77 non-clinical  High BPD features *n* = 30  Low BPD features (controls) *n* = 47 | Schedule for Nonadaptive and Adaptive Personality (SNAP-2; Simms & Clark, 2006) | Facial trust appraisal after positive, negative, or neutral affective priming | BPD participants show more untrustworthiness bias, i.e., lower trust ratings regardless of priming. Trust appraisal influenced by negative priming in BPD more than controls. |  |
| Fertuck et al. (2013) | Trust appraisal | *N* = 36  BPD = 17  Control = 19 | SCID-II | Fear and trust facial appraisal | BPD participants show an increased response bias in trustworthiness appraisal compared with control (no significant differences in sensitivity and discriminability). |  |
| Nicol et al. (2013) | Trust appraisal | Total *N* = 40 females  BPD *n* = 20  Control *n* = 21 | SCID-II | Childhood Trauma Questionnaire (CTQ)  Facial trust appraisal | BPD group judged faces as being less trustworthy. Furthermore, there was a correlation between CTQ and bias only in the BPD group. |  |
| Houben et al. (2018) | Trust appraisal | *N =* 58  BPD *n*= 30  Control *n*= 28 | Assessment of DSM-IV Personality  Disorders-Borderline scale (ADP-IV- Borderline scale (Schotte et al., 1998) | Trust appraisal of others in daily life | Lower appraisal of others’ trustworthiness in daily life in BPD participants. |  |
| Fertuck et al. (2019) | Trust appraisal | *N =* 33  BPD *n* = 16  Control *n* = 17 | SCID-II | Fear and trust appraisal | Lower trustworthiness appraisal in BPD participants.  Lower BOLD activity while trustworthiness appraisal in prefrontal cortex related to bias intensity |  |
| Biermann et al. (2022) | Trust appraisal | *N =* 149 females  BPD *n* = 75  Control *n* = 67 | International  Personality Disorder Examination (IPDE; Loranger, 1997) | Trust appraisal of faces with and without face masks | BPD participants show an untrustworthiness bias, i.e., lower trust ratings regardless of presence of face mask |  |
| King-Casas et al. (2008) | Behavioral manifestations | *N* = 93  BPD *n* = 55  Controls *n* = 38 | DIPD-IV | Multiple round trust game | BPD participants more likely to cause cooperation ruptures  BPD participants sustain lower rates of coaxing behaviors |  |
| Unoka et al. (2009) | Behavioral Manifestations | *N* = 75  BPD *n* = 25  Healthy controls *n* = 25  Major depression controls *n* = 25 | ZAN-BPD | Single trust game  Risk lottery game | BPD participants transferred less money during the trust game: untrustworthiness bias.  No behavioral differences in the lottery game. |  |
| Saunders et al. (2016) | Behavioral manifestations | *N* = 40 females  BPD *n* = 20  Controls *n* = 20 | IPDE (Loranger, 1997) | Iterated form of the prisoner’s dilemma | Only BPD participants failed in building cooperative relationships. |  |
| Hepp et al. (2016) | Behavioral manifestations | *N* = 52  BPD *n* = 26  Healthy controls *n* = 26 | IPDE (Loranger, 1997) | Dictator game | No differences between BPD and controls in money shared. |  |
| Lévay et al. (2021) | Behavioral manifestations | *N* = 60  BPD *n* = 30  Healthy controls *n* = 30 | SCID-II | Slider Measure of Social Value Orientation –Self-to-Other  Condition and Other-to-Self Condition | No differences between BPD and controls in money shared. BPD participants reported more selfish expectations about how others would share money with them. |  |
| Niedtfeld & Kroneisen (2020). | Behavioral manifestations | *N* = 100  Females BPD *n* = 51  Healthy controls *n* = 50 | IPDE (Loranger, 1997) | Single round trust game | BPD participants transferred less money during the trust game only when playing with trustworthy faces (no significant differences when playing with untrustworthy faces). |  |
| Franzen et al. (2011) | Trust learning | *N* = 60  BPD *n* = 30  Healthy control *n* = 30 | SCID-II | Multi-round trust-game | Both BPD participants and control participants adapted their investment behaviors according to counterparts’ facial expressions. |  |
| Bo et al. (2017) | Trust learning | *N =* 25  BPD *adolescents (age 13-17)* | - Clinical evaluation  - BPFS-C (Crick et al., 2005) | Inventory of Parent and Peer Attachment—Revised (IPPA-R, Gullone & Robinson, 2005) | Pre-post changes (after MBT group treatment) in trust toward parents and peers. |  |
| Fineberg et al. (2018) | Trust learning | *N =* 43  BPD *n* = 20  Controls *n* = 23 | - SCID-II  - Borderline Symptom List 23 (BSL-23; Bohus et al., 2009). | Reward learning task. | BPD participants had lower trust learning scores even if they weighted more social cues compared with controls. |  |
| Abramov et al., (2020) | Trust learning | *N* = 234 non-clinical | McLean Screening Instrument for BPD  (MSI-BPD; Zanarini et al., 2003). | Multi-round trust-game | Individuals with a higher number of BPD traits show a greater decline in investments during the trust-formation phase. |  |
| Abramov et al., (2022) | Trust learning | *N* = 234 non-clinical | McLean Screening Instrument for BPD  (MSI-BPD; Zanarini et al., 2003). | Multi-round trust-game | BPD effects on investments partly explained by feelings of rejection and self-protective beliefs. |  |
|  |  | | | | | |
